# Cloning, Soluble Expression and Purification of High Yield Recombinant hGMCSF in *Escherichia coli*

**DOI:** 10.3390/ijms12032064

**Published:** 2011-03-22

**Authors:** Krishna M.P. Das, Sampali Banerjee, Nivedita Shekhar, Karpagavalli Damodaran, Rahul Nair, Sandeep Somani, Veena P. Raiker, Shweta Jain, Sriram Padmanabhan

**Affiliations:** 1 Clone Development Team, Lupin Limited, Biotechnology R & D, Gat #1156, Ghotawade Village, Mulshi Taluka, Pune-411042, India; E-Mails: krishnap@lupinpharma.com (K.M.P.D.); sampalibanerjee@lupinpharma.com (S.B.); 2 Mammalian Bioassay Team, Lupin Limited, Biotechnology R & D, Gat #1156, Ghotawade Village, Mulshi Taluka, Pune-411042, India; E-Mail: niveditashekhar@lupinpharma.com; 3 Analytical development Team, Lupin Limited, Biotechnology R & D, Gat #1156, Ghotawade Village, Mulshi Taluka, Pune-411042, India; E-Mails: karpagavallid@lupinpharma.com (K.D.); veenaraiker@lupinpharma.com (V.P.R.); shwetajain@lupinpharma.com (S.J.); 4 Upstream Development Team, Lupin Limited, Biotechnology R & D, Gat #1156, Ghotawade Village, Mulshi Taluka, Pune-411042, India; E-Mail: rahulnair@lupinpharma.com; 5 Downstream Development Team, Lupin Limited, Biotechnology R & D, Gat #1156, Ghotawade Village, Mulshi Taluka, Pune-411042, India; E-Mail: sandeepsomani@lupinpharma.com; 6 Biotechnology R&D, Lupin Limited, Biotechnology R & D, Gat #1156, Ghotawade Village, Mulshi Taluka, Pune-411042, India

**Keywords:** human granulocyte macrophage colony stimulating factor, on-column cleavage, TRX fusion, IMAC, enterokinase

## Abstract

Expression of human granulocyte macrophage colony stimulating factor (hGMCSF), a cytokine of therapeutic importance, as a thioredoxin (TRX) fusion has been investigated in *Escherichia coli* BL21 (DE3) codon plus cells. The expression of this protein was low when cloned under the T7 promoter without any fusion tags. High yield of GMCSF was achieved (∼88 mg/L of fermentation broth) in the shake flask when the gene was fused to the *E. coli* TRX gene. The protein was purified using a single step Ni^2+^-NTA affinity chromatography and the column bound fusion tag was removed by on-column cleavage with enterokinase. The recombinant hGMCSF was expressed as a soluble and biologically active protein in *E. coli*, and upon purification, the final yield was ∼44 mg/L in shake flask with a specific activity of 2.3 × 10^8^ U/mg. The results of Western blot and RP-HPLC analyses, along with biological activity using the TF-1 cell line, established the identity of the purified hGMCSF. In this paper, we report the highest yield of hGMCSF expressed in *E. coli*. The bioreactor study shows that the yield of hGMCSF could be easily scalable with a yield of ∼400 mg/L, opening up new opportunities for large scale production hGMCSF in *E. coli*.

## 1. Introduction

Human granulocyte macrophage colony stimulating factor (hGMCSF) is a cytokine, secreted by macrophages, T cells, mast cells, endothelial cells and fibroblasts in response to immune and inflammatory stimuli. Mature human GMCSF is a glycoprotein and consists of 127 amino acid residues, with four cysteines being involved in two disulfide bonds [1]. Human GMCSF is an important therapeutic cytokine used in the treatment of myeloid leukemia, neutropenia and aplastic anemia [2]. Many attempts have been undertaken to synthesize biologically active recombinant hGMCSF; however, transfected mammalian cells are not preferred as an expression system for producing GMCSF for biological and structural studies due to low expression levels and the presence of contaminating CSFs secreted by the mammalian cells themselves [3]. This problem can be handled using an *Escherichia coli* expression system to produce large quantities of recombinant protein. *E. coli* has widely been used for recombinant protein production [4] due to its ability to grow rapidly and at high density on inexpensive substrates, combined with its well-characterized genetics. A variety of cloning and expression vectors, recombinant fusion tags and mutant strains are available for commercial manufacture of recombinant proteins [5,6]. Although attractive, some potential disadvantages of this expression system include lack of post translational modifications [7], lack of the proper secretion system for efficient release of produced protein into the growth medium, inefficient cleavage of amino terminus methionine resulting in lower protein stability and increased immunogenicity together with the limited ability to facilitate extensive disulfide bond formation and improper folding resulting in inclusion body formation [8].

Protein misfolding or inaccurate processing by cellular molecular chaperones ultimately results in formation of biologically inactive protein. Hence, optimization of the expression conditions or laborious refolding studies is required to achieve an active protein. Also, many eukaryotic proteins cannot be expressed successfully in *E. coli*, and the conventional method to express such proteins is as fusion tags [9,10]. There have been reports of expression of hGMCSF as intein fusion entities [11] and GCSF fusion proteins [12], with all of them being expressed as insoluble protein aggregates. Soluble protein production in *E. coli* is still a major bottleneck for investigators, and a couple of efforts have been reported to improve the solubility or folding of recombinant protein produced in *E. coli* [13]. These include strategies like co-expression of chaperone proteins such as GroES, GroEL, DnaK and DnaJ, lowering incubation temperature, use of weak promoters, addition of sucrose and betaine in the growth media, use of richer media with phosphate buffer such as terrific broth (TB), use of signal sequence to export the protein to the periplasmic fraction and use of fusion tags to aid in expression and protein purification [9]. A number of fusion tags are available for the ease of expression and purification of recombinant proteins [14,15] and mostly they promote purification of the fused protein, though some of them (thioredoxin, NusA, *etc*.) are also reported to increase the solubility of the target proteins in comparison to unfused proteins when overexpressed in *E. coli* [16]. In this paper, we describe the overexpression of hGMCSF as a soluble thioredoxin (TRX)-fusion and purification to homogeneity with very high yield after removal of the fusion tag by enterokinase digestion.

## 2. Results and Discussion

### 2.1. Cloning and Expression of hGMCSF

**Figure 1.**
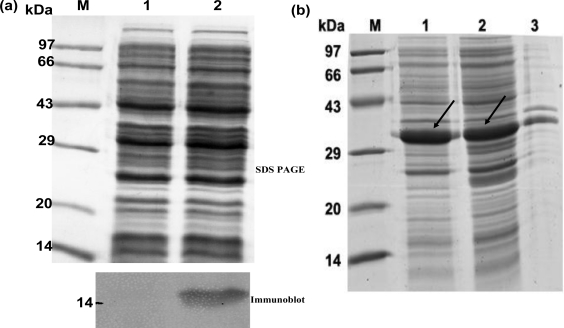
Expression analysis of pET21a-hGMCSF and pET32a-hGMCSF. (**a**) Upper Panel: SDS-PAGE analysis of pET21a-hGMCSF expression in BL21 DE3 codon plus cells with IPTG induction, showing absence of visible expression. Lane M: molecular weight Marker; lane 1: Empty vector control; lane 2: expression from pET21-hGMCSF. Lower Panel: Immunoblot using mouse monoclonal anti-hGMCSF antibody showing low expression level of hGMCSF; (**b**) SDS-PAGE analysis of TRX-hGMCSF fusion protein expression. The pET32a-hGMCSF construct was transformed into BL21 (DE3) codon plus cells. The cells were induced with 1 mM IPTG for 3 h. Lane M: Molecular weight Marker; lane 1: Crude cell lysate; lane 2: Soluble fraction and lane 3: Insoluble fraction. Arrows indicate TRX-GMCSF protein.

**Figure 2.**
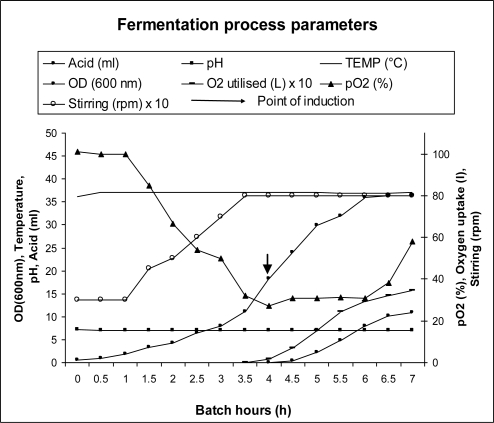
Expression of TRX-hGMCSF in 2 L fermenter in In-house medium. Various fermentation parameters were plotted against the batch hour. The cells were induced with 1 mM IPTG at an OD_600_ of 18 and the culture was grown for another 3 h until it reached an OD_600_ of 37.

hGMCSF was cloned in pET21a and expressed in BL21 (DE3) codon plus cells in the shake flask scale (100 mL LB). As seen in Figure 1a, there was no visible expression of recombinant hGMCSF by SDS-PAGE (upper panel, lane 2) and the expression was evident only after immunoblot analysis (lower panel, lane 2). As the poor expression of GMCSF was unsuitable for any further experimentation, the gene was cloned as a TRX fusion and the expression was carried out in the same cell line as described before. The results shown in Figure 1b indicate that the expression of TRX-GMCSF in soluble form (Figure 1b, lane 2). Bioreactor study on a 2 L scale (Figure 2) was carried out using in-house medium and the total protein in the soluble fraction was found to be 4.95 g/L, which corresponds to nearly a yield of 400 mg of crude GMCSF protein/L of fermentation medium.

### 2.2. Purification of TRX-hGMCSF Followed by Separation of hGMCSF from the Fusion Tag

**Figure 3.**
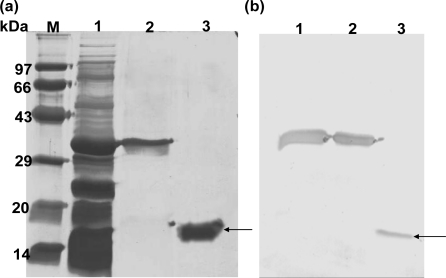
SDS-PAGE and Western blot analysis of purified hGMCSF. Soluble fraction containing TRX-hGMCSF was passed through Ni-NTA column and the fractions containing pure TRX-GMCSF were dialyzed and bound to Ni-NTA a second time in batch mode. The fusion protein was cleaved in the bound state with enterokinase enzyme in the presence of 1 mM CaCl_2_. The flow through containing hGMCSF was collected and analyzed. (**a**) SDS-PAGE showing different purification steps. Lane M: Molecular weight Marker; lane 1: Soluble fraction containing TRX-hGMCSF fusion; lane 2: Purified TRX-GMCSF fusion, lane 3: purified hGMCSF; (**b**) Immunoblot using mouse monoclonal anti-hGMCSF antibody to confirm the identity of hGMCSF. Lane 1: Soluble fraction containing TRX-hGMCSF fusion; lane 2: Purified TRX-GMCSF fusion; lane 3: purified hGMCSF. Arrows represent the purified hGMCSF in both SDS-PAGE and immunoblot.

**Figure 4.**
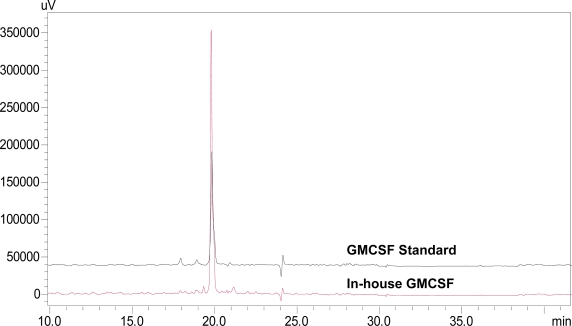
Reverse phase chromatograph showing comparison of standard GMCSF and in-house purified hGMCSF with optimum mobile phase consisting of 0.1% TFA in 10% acetonitrile (**a**) and 0.1% TFA in 90% acetonitrile (**b**). Flow rate was maintained at 1 mL/min and detection was at 215 nm. Black line represents standard hGMCSF while red line indicates the in-house purified rhGMCSF.

**Figure 5.**
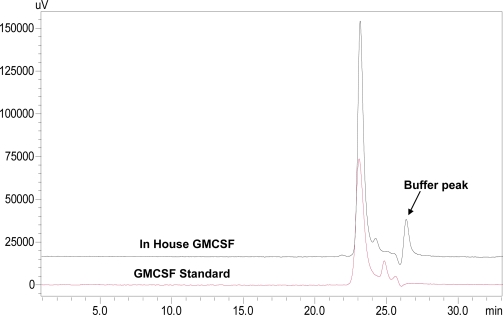
Size Exclusion HPLC profile of standard and in-house purified GMCSF preparations. Size exclusion chromatograph showing comparison of standard GMCSF and in-house purified hGMCSF with optimum mobile phase consisting of 1.15 g di-sodium hydrogen phosphate, 0.2 g of potassium hydrogen phosphate and 23.4 g of sodium chloride. Flow rate was maintained at 0.5 mL/min and detection was at 215 nm. Black line represents standard hGMCSF while red line indicates the in-house purified rhGMCSF.

**Figure 6.**
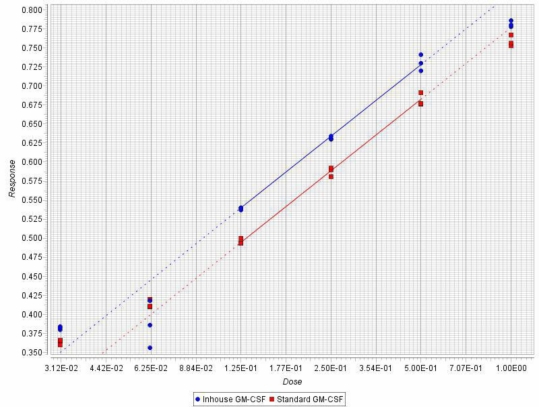
The biological activity of in-house hGMCSF was assessed using TF-1 cell proliferation assay. The activity data was analyzed statistically using Parallel Line Assay software (PLA 2.0). The doses are indicated on the horizontal axis, whereas the corresponding responses are represented on the vertical axis. The individual responses for each treatment are symbolized by the red squares for the standard preparation and by the blue circles for the sample preparation.

The TRX-GMCSF, containing a six His-tag in between the fusion partners TRX and GMCSF, was purified through Ni^2+^-NTA sepharose following the protocol described in the Experimental section. The purified fusion protein (Figure 3a, lane 2) after enterokinase cleavage and second round of purification yielded >95% pure hGMCSF protein (Figure 3a, lane 3) with a final yield of ∼44 mg/L and a fold purification of 2.5 (Table 1). Immunoblot analysis with mouse monoclonal anti-hGMCSF antibody confirmed the identity of the purified protein, which has a theoretical molecular mass 14.4 kDa (Figure 3b). The purity of the purified soluble GMCSF from the above fusion tag clone following the described method was analyzed by RP-HPLC and SE-HPLC for identity and similarity study with commercial GMCSF (Sigma, U.S.). RP-HPLC profiles of both soluble hGMCSF and commercial hGMCSF showed a similar pattern (Figure 4) at a retention time of 19.797 min with a purity of ∼95%, which is better than the commercial protein (∼90.2%), indicating the efficient separation and purification of the protein of interest. The commercial hGMCSF used was procured from Sigma (G 5035) and the product is supplied as a lyophilized powder from a 10 mM sodium citrate solution, pH 3.5, with no other proteinous material. This was also evident from the profile, with the absence of any major peak other than hGMCSF peak. The SE-HPLC analysis was carried out to determine the presence of GMCSF related impurities like aggregation and different conformational forms [17]. The chromatogram (Figure 5) shows that the in-house purified hGMCSF is ∼92% pure with no detectable aggregation or other conformational forms, while purity of the commercial GMCSF preparation was found to be relatively less (∼86.8%). The biological activity assay data indicate that the in-house hGMCSF is more active (potency 1.396) than the commercial preparation (Figure 6) and this could be partially attributed to the better purity of the in-house protein preparation.

For structural functional and clinical studies, therapeutic proteins in soluble active forms are in large demand. Human GMCSF protein has been described to function in the treatment of myeloid leukemia, neutropenia and aplastic anemia. Although, different expression systems have been explored to express recombinant human GMCSF (such as CHO, yeast, bacteria, *etc*.), all of them have certain degrees of limitations. It has been reported that deglycosylated hGMCSF is at least 20-fold more active than its glycosylated variant expressed in CHO cells [18–20]. Similarly, *Saccharomyces cerevisae* derived GMCSF is clinically unsuitable due to varying degrees of glycosylation [21]. On the other hand, hGMCSF expressed in *E. coli* has been found to have similar biological activity to the native protein [22], indicating the non-essentiality of glycosylation for bioactivity of the GMCSF protein.

*E. coli* expression system offers several advantages like high expression level, rapid growth, simple media requirement, *etc*. Recombinant human GM-CSF produced in *E. coli* ends up in inclusion bodies (IBs) and has certain drawbacks, including complex processing, low specific activity, and poor *in vitro* renaturation [23]. Recently, hGMCSF has been reported to be expressed in *E. coli* BL21 (DE3) cells without IPTG induction as insoluble aggregates [1]. The protein has been purified after solubilization and the final yield was found to be ∼44 mg/L. Also, intein fusion of hGMCSF has been reported in the recent past in *E. coli* [11] as well as in *Pichia* [24]. However, hGMCSF expression as a soluble protein in *E. coli* is host dependent, and in both the cases, authors have used dithiothreitol (DTT) to remove the fusion tag. Since DTT concentrations above 30 mM are known to destabilize the disulfide bonds [25] and use of DTT to remove the fusion tag could hamper the two disulfide bonds that are crucial for hGMCSF activity [1], use of DTT in purification of GMCSF using such a strategy appears tricky and challenging.

High GC content and the presence of rare codons in the native human GMCSF gene are reported to be causative hurdles in the expression of recombinant human GMCSF (rhGMCSF) in *E. coli* [26]. In order to achieve better expression in *E. coli*, we have reduced the GC content of the hGMCSF gene at the 5′ terminus and also used BL21 (DE3) codon plus cells for expression studies to supply rare codons required for efficient and optimal expression of the protein.

Here, we report the soluble expression of hGMCSF in *E. coli* and on-column cleavage and removal of the TRX fusion tag from hGMCSF for the first time. By following the process described in this article, we achieved ∼95% pure rhGMCSF protein with a specific activity of 2.3 × 10^8^ U/mg with a potency of 1.396 as evident from the statistical analysis using the Parallel Line Assay (PLA) software. The yield of hGMCSF to ∼44 mg/L with a recovery of ∼46 % observed in the present study is the highest to date [11]. Although fusion tags like intein (55 kDa) have been reported for GMCSF fusions [11], the use of TRX as a fusion tag as described in this paper has an additional advantage. It offers higher molar yield of the protein of interest after tag removal since the size of the TRX tag is relatively smaller (20 kDa). Our methodology of obtaining soluble GMCSF using the procedure as mentioned avoids the cumbersome procedures of refolding and purification of GMCSF from bacterial inclusion bodies, making the proposition attractive and user-friendly. Moreover, the expression of hGMCSF from the present construct in a bioreactor at 2 L scale yielded ∼400 mg/L; thus presenting a promising cost-effective alternative for obtaining GMCSF protein in manufacturing scale.

## 3. Experimental Section

### 3.1. Cloning of hGMCSF in pET21a Vector and in pET32a as a TRX Fusion Tag

The hGMCSF gene was amplified using a synthetic gene (GenScript, U.S.) by polymerase chain reaction using the oligos with reduced GC content, forward: 5′ CCG CCG GAA TTC GGA TCC GAT GAT GAT GAT AAA GCA (GGC) CCC (ACT) GCC (GTG) CGC (GCC) TCG (TGC) CCC (AGC) AGC (ATC) 3′ as and reverse: 5′ CCG CCG GAA TTC AAG CTT TCA CTC CTG GAC TGG CTC CCA 3′. The codons within brackets show the original sequence of the human GMCSF gene. PCR cycling conditions were: 94 °C for 4 min followed by 5 cycles of 94 °C for 30 s, 53 °C for 30 s, 72 °C for 30 s and 25 cycles of 94 °C for 30 s, 65 °C for 30 s, 72 °C for 30 s. The PCR product was cloned into pET21a as an *Nde*I/*Hin*dIII fragment and into pET32a (Novagen, U.S.) as a *BamH*I/*Hin*dIII fragment and all the clones were confirmed by DNA sequencing.

### 3.2. Expression of pET21a-rhGMCSF and pET32a-rhGMCSF in Shake Flask

The pET21a-rhGMCSF and pET32a-rhGMCSF constructs were separately introduced into BL21 (DE3) codon plus cells and expression was carried out at 37 °C for 4 h in 100 mL Luria Broth containing 100 μg/mL ampicillin. The cells were induced with 1 mM IPTG and the induced cell pellet was suspended in 10 mM TrisCl, pH 8.0 followed by lysis by sonication (Sonics Vibracell, U.S.). Separation of soluble and insoluble fractions was carried out by centrifugation of the sonicated lysate at 13,000 rpm for 10 min and both the fractions were analyzed on SDS-PAGE followed by Coomassie blue staining.

### 3.3. Bioreactor Studies

The large scale fermentation was carried out in a 2 L bioreactor (Sartorius, Germany) with 2 L in-house media with 1% glycerol [27]. 2% of the overnight culture was used as inoculum and the culture was grown at 37 °C and pH 7.0 up to an OD_600_ of 18. The cells were induced with 1 mM IPTG and the culture was grown for another 3 h until it reached an OD_600_ of 37. The culture media was centrifuged at 8000× g for 10 min and the induced cell pellet was resuspended in 10 mM TrisCl pH 8.0. The suspension was subjected to cell disruption using a high pressure homogenizer (M/S Niro Soavi, Italy) at 800 to 900 bars for two passages. The homogenized cell lysate was centrifuged at 12,500× g for 15 min at 4 °C to separate the soluble and insoluble fractions.

### 3.4. Purification hGMCSF Using Ni^2+^-NTA Column

The cleared soluble fraction containing TRX-rhGMCSF was passed through Ni^2+^-NTA column (GE Healthcare, Sweden) pre-equilibrated with 10 mM TrisCl, pH 8.0 containing 10 mM imidazole (Sigma, U.S.). After washing the column with equilibration buffer, the bound protein was eluted with a gradient of imidazole (0.1–0.5 M). The elute fractions containing the majority of the pure TRX-hGMCSF protein was dialyzed overnight against 10 mM TrisCl, pH 8.0 at 4 °C. For enterokinase reaction, pure TRX-hGMCSF was passed through a second round of purification on Ni^2+^-NTA Sepharose that was pre-equilibrated with TrisCl, pH 8.0 in a batch mode in continuous motion in a rotating mixer. The bound fusion protein, was digested with bovine enterokinase (4.5 U/mg of pure fusion protein) (Novagen, U.S.) for four hours at room temperature in the presence of 1 mM CaCl_2_. The flow through was collected by centrifugation of the contents at 4400 rpm for 10 min and all samples were analyzed by SDS-PAGE followed by silver staining.

### 3.5. Characterization of rhGMCSF by RP-HPLC

The RP-HPLC was carried out using an ACE C18 (4.6 mm × 150 mm) column on SHIMADZU LC-2010C_HT_ HPLC system provided with a quaternary pump, a thermostatted autosampler, a thermostatted column compartment, and a multiple wavelength ultraviolet (UV) detector. Data was collected and analyzed using LC Solution Software (Version 1.24). The mobile phase consisted of 0.1% TFA in 10% Acetonitrile (A) and 0.1% TFA in 90% Acetonitrile (B). The system was equilibrated with a mixture A–B (90:10) until a stable baseline was obtained. Separations were performed using a stepwise gradient in the following manner: from 10% to 65% mobile phase B over a period of 20 min, followed by 65% to 100% mobile phase B over a period of 3 min. The flow-rate was maintained at 1.0 mL/min with detection at 215 nm at 30 °C.

### 3.6. Characterization of rhGMCSF by SE-HPLC

SEC was performed with SHIMADZU LC-2010C_HT_ HPLC system provided with a quaternary pump, a thermostatted autosampler, a thermostatted column compartment, and a multiple wavelength ultraviolet (UV) detector. Data was collected and analyzed using LC Solution Software (Version 1.24). A TSK-GEL G3000SWXL 300 mm × 7.8 mm column (MW range: 1000–500,000 Da) (Tosoh Bioscience LLC, Montgomeryville, PA, U.S.) was chosen for the present studies. . The optimal mobile phase composition consisted of 1.15 g Di-sodium hydrogen phosphate, 0.2 g of Potassium hydrogen phosphate and 23.4 g of sodium chloride. The detector was set at 215 nm and the flow rate at 0.5 mL/min.

### 3.7. Western Blot Analysis

Protein samples were separated on denaturing SDS-PAGE and transferred to nitrocellulose membrane. Immunoblot was performed using mouse monoclonal anti-hGMCSF antibody (Santacruz, U.S.) followed by goat anti-mouse secondary antibody (Bangalore Genei, India). The blot was developed using the substrate BCIP/NBT.

### 3.8. Bioassay for hGMCSF

TF-1 cells were maintained in RPMI-1640 with 10% FBS and 2 ng/mL rhGMCSF at 37 °C in 5% CO_2_. The cells were starved for 14–16 h in RPMI-1640 with 2.5% FBS. After starvation, the cells were plated in RPMI-1640 with 5% FBS at a seeding density of 1 × 10^4^ cells/well/50 μL. Standard and samples were added (50 μL/well) at different concentrations and the plate was incubated for 48 h at 37 °C in 5% CO_2_. To each well, 20 μL of MTS was added and amounts of formazan formed (an indicator of number of live cells, *i.e*., biological activity) were estimated by measuring OD_490_ after an additional 4 h of incubation. The ED_50_ value was determined and one unit of activity is defined as reciprocal of ED_50_. The data was analyzed statistically using Parallel Line Assay software (PLA 2.0), which uses three tests for validity of the assay: test of regression, test of linearity and test of parallelism. This analysis gives potency ratio that expresses the potency of the unknown sample in comparison to the potency of the standard. The graph was obtained by plotting responses against doses.

## 4. Conclusions

In this article, we report for the first time the hyper expression of hGMCSF in *E. coli* at shake flask level with a very high yield (∼44 mg/L) which was easily scalable to ∼400 mg/L in a bioreactor. Such a strategy of expressing rhGMCSF demonstrates the possibility of achieving high yield therapeutic proteins and could be applied to other therapeutic proteins.

## Figures and Tables

**Table 1.** Summary of purification of hGMCSF expressed as TRX fusion from 100 mL flask culture.

**Fraction**	**Volume (mL)**	**Protein (mg/mL)**	**Total Protein (mg)**	**Total Activity (U)**	**Specific Activity (U/mg)**	**Fold Purification**	**%Yield**
Crude cell lysate	10	2.39	23.9	2.19 × 10^8^	9.16 × 10^6^	1.0	100
Purified TRX-GMCSF fusion	20	0.43	8.6	11.24 × 10^7^	1.3 × 10^7^	1.42	51
Purified GMCSF	20	0.22	4.4	10.20 × 10^7^	2.3 × 10^7^	2.5	46
